# Changes in retail food environments around schools over 12 years in Flanders, Belgium

**DOI:** 10.1093/eurpub/ckac129.417

**Published:** 2022-10-25

**Authors:** V Smets, S Vandevijvere

**Affiliations:** Lifestyle and Chronic Diseases, Sciensano, Anderlecht, Belgium

## Abstract

**Background:**

Food environments influence food preferences, in particular among children. Besides their homes, children spend most of their time in and around the school. This study mapped changes in retail food environments around primary and secondary schools in Flanders between 2008 and 2020. In addition, associations between those indicators and children's weight status were assessed.

**Methods:**

The food environment near primary and secondary schools was mapped using three spatial indicators: 1) The density of different types of food retailers within 1000m road distance from the school entrance, 2) the percentage of schools with at least one food retailer of a certain type within 1000m road distance from the entrance, and 3) the median walking distance from the school entrance to the nearest food retailer of a certain type. Associations between the density of convenience stores, as well as fast food outlets around the schools and the weight status of the schools’ children were assessed using generalized linear models adjusted for level of urbanization, socio-economic status of children at school level and sex.

**Results:**

Food environments near schools in Flanders were found to be unhealthy, with a significant increase in fast-food outlets and convenience stores between 2008 and 2020. Food environments near schools with a higher proportion of children from a poor socio-economic background were found more unhealthy than those near schools with a lower proportion of such children, regardless of the urbanization level. A significant positive association was found between the density of fast food outlets as well as convenience stores around primary schools and the weight status of children aged <6 years and 6-12 years.

**Conclusions:**

Food environments around schools in Flanders became more unhealthy over time and were associated with children's weight status. The government therefor has the responsibility to create healthy food environments near schools to protect children's health.

**Key messages:**

• The food environment near schools became more unhealthy between 2008 and 2020.

• The food environment near schools was associated with younger children's weight status.

